# Three marine species of the genus *Fulvivirga,* rich sources of carbohydrate-active enzymes degrading alginate, chitin, laminarin, starch, and xylan

**DOI:** 10.1038/s41598-023-33408-4

**Published:** 2023-04-18

**Authors:** Tra T. H. Nguyen, Tien Q. Vuong, Ho Le Han, Zhun Li, Yong-Jae Lee, Jaeho Ko, Olga I. Nedashkovskaya, Song-Gun Kim

**Affiliations:** 1 Biological Resource Center, Korean Collection for Type Cultures, Korea Research Institute of Bioscience and Biotechnology, Jeongeup, 56212 Republic of Korea; 2 Department of Biotechnology, KRIBB School, University of Science and Technology (UST), Daejeon, 34113 Republic of Korea; 3 Hanoi University of Science, Vietnam National University, Hanoi, 10000 Vietnam; 4 The University of Danang, University of Science and Technology, 54 Nguyen Luong Bang St., Da Nang, 550000 Vietnam; 5 G.B. Elyakov Pacific Institute of Bioorganic Chemistry of the Far-Eastern Branch of the Russian Academy of Sciences, Vladivostok, Russia 690022

**Keywords:** Biotechnology, Microbiology

## Abstract

*Bacteroidota* is a group of marine polysaccharide degraders, which play a crucial role in the carbon cycle in the marine ecosystems. In this study, three novel gliding strains, designated as SS9-22^T^, W9P-11^T^, and SW1-E11^T^, isolated from algae and decaying wood were proposed to represent three novel species of the genus *Fulvivirga*. We identified a large number of genes encoding for carbohydrate-active enzymes, which potentially participate in polysaccharide degradation, based on whole genome sequencing. The 16S rRNA sequence similarities among them were 94.4–97.2%, and against existing species in the genus *Fulvivirga* 93.1–99.8%. The complete genomes of strains SS9-22^T^, W9P-11^T^, and SW1-E11^T^ comprised one circular chromosome with size of 6.98, 6.52, and 6.39 Mb, respectively; the GC contents were 41.9%, 39.0%, and 38.1%, respectively. The average nucleotide identity and the digital DNA-DNA hybridization values with members in the genus *Fulvivirga* including the isolates were in a range of 68.9–85.4% and 17.1–29.7%, respectively, which are low for the proposal of novel species. Genomic mining in three genomes identified hundreds of carbohydrate-active enzymes (CAZymes) covering up to 93 CAZyme families and 58–70 CAZyme gene clusters, exceeding the numbers of genes present in the other species of the genus *Fulvivirga.* Polysaccharides of alginate, chitin, laminarin, starch, and xylan were degraded in vitro, highlighting that the three strains are rich sources of CAZymes of polysaccharide degraders for biotechnological applications. The phenotypic, biochemical, chemotaxonomic, and genomic characteristics supported the proposal of three novel species in the genus *Fulvivirga*, for which the names *Fulvivirga ulvae* sp. nov. (SS9-22^T^ = KCTC 82072^T^ = GDMCC 1.2804^T^), *Fulvivirga ligni* sp. nov. (W9P-11^T^ = KCTC 72992^T^ = GDMCC 1.2803^T^), and *Fulvivirga maritima* sp. nov. (SW1-E11^T^ = KCTC 72832^T^ = GDMCC 1.2802^T^) are proposed.

## Introduction

Degradation of marine polysaccharides by heterotrophic bacteria plays an important role in the carbon cycle^1,2^. Polysaccharides are long-chain polymeric carbohydrate molecules constructed by glycosidic linkages that connect monosaccharide units^3^. In the marine environment, marine algae are one of the main producers of polysaccharides on a global scale. Red algae, such as *Eucheuma* sp.^4^ and *Polyneura* sp.^5,6^, contain agar, carrageenan, mannan, and xylan. Green algae, such as *Chlamydomonas* sp.^7^, *Chlorella* sp., and *Ulva* sp.^8,9^, contain cellulose, sulfated galactans, ulvane, and xylan. Brown algae, such as *Ascophyllum* sp., *Fucus* sp.^10^, and *Laminaria* sp.^11^, contain alginate, fucoidan, and laminarin. Diatom algae, such as *Tetraselmis* sp.^12^, contain arabinogalactan, fucose-containing sulfated polysaccharides, mannan, and galacturonan^13^. In marine polysaccharides, the glycan backbone usually holds substitutions of the methyl group^14^, pyruvate^15^, and sulfate^16^ for marine organisms to adapt to the marine conditions^17,18^. Marine heterotrophic bacteria have various enzymes to digest these polysaccharides by breaking the glycosidic bonds, and to convert the high molecular weight compounds into lower molecular weight compounds^19^. This production and biodegradation of polysaccharides is considered a critical step of the carbon cycle in marine ecosystems^13,20,21^. On the other hand, algal oligosaccharides have many potential applications in functional food, biomedicine and cosmetics^22^, and biofuel and pulp industries^23,24^. For instance, laminarin and laminarin oligosaccharides were demonstrated to have various biological activities, including antioxidant, antitumor, and prebiotic effects and to contribute to the immunomodulatory mechanism^25^. Furthermore, alginate and its derived oligosaccharides also have similar activities such as antimicrobial, antihypertensive, anticoagulant, and antidiabetic activities^26,27^. The demand for bio-production of algal oligosaccharides is accordingly increasing. It is thus important to identify novel polysaccharide-degrading microorganisms.

The phylum *Bacteroidota* (a heterotypic synonym of *Bacteroidetes*) contains unique genes for polysaccharide degradation^28^. This unique machinery comprises SusD, which captures the polysaccharides. Extracellular carbohydrate-active enzymes (CAZymes) are then secreted to degrade the polysaccharides into oligosaccharides, which are imported to the periplasm via SusC transporters on the membrane^29,30^. In the periplasm, sugar-degrading enzymes further degrade the oligosaccharides into monosaccharides^31^. Subsequently, dedicated transporters deliver these monosaccharides to cross into the cytoplasm^29,31,32^. The regulators for gene expression are operated by sensing the degraded small molecules that are products of polysaccharide degradation^33^. These CAZymes, transporters, and regulators are closely encoded by the region of a chromosome known as polysaccharide utilization loci (PUL)^34^. In the marine environment, the representatives of the class *Flavobacteriia* of the phylum *Bacteroidota*, which are well known as degraders of marine polysaccharides^35–38^, contain PULs with a high number of sulfatases^36^. Sulfatases are required to remove the sulfate esters or sulfamates from these sulfated polysaccharides^39^, which undergo sulfation under a high concentration of sulfate in the marine environment. However, few studies have reported on the polysaccharide degradation capacity of the class *Cytophagia,* particularly the family *Fulvivirgaceae.*

Members of the genus *Fulvivirga*, which belong to the phylum *Bacteroidota,* have been discovered in various areas of the marine environment^40–45^, but their ability to degrade polysaccharides is not well understood. The genus *Fulvivirga* first described by Nedashkovskaya et al*.* (2007) belongs to the family *Fulvivirgaceae*, order *Cytophagales,* and class *Cytophagia.* Members of the genus *Fulvivirga* are heterotrophic, Gram-staining-negative, non-flagellated, non-spore-forming, rod-shaped cells, and share menaquinone 7 (MK-7) as the major respiratory quinone. At the time of writing, the genus consists of seven species, including *F. kasyanovii*^40^, *F. imtechensis*^41^, *F. lutimaris*^43^, *F. aurantia*^44^, *F. lutea*^42^, *F. marina*^45^, and *F. sediminis*^45^. Thus far, members of this genus are reported to degrade starch only among polysaccharides^40,42–44^.

In this study, we isolated three strains that degrade polysaccharides and propose three novel species of the genus *Fulvivirga* with the type strains SS9-22^T^, W9P-11^T^, and SW1-E11^T^, based on a comparative and comprehensive characterization of the isolates with the seven other species in the genus *Fulvivirga*. The complete whole-genome sequences of the three strains were determined and the repertoire of CAZymes and PULs was analyzed. The capability of polysaccharide degradation of the isolates was studied in silico and in vitro. The presence of abundant CAZymes and the ability to degrade polysaccharides indicate that the three novel strains are rich sources of carbohydrate-active enzymes for degradation of polysaccharides and potential biotechnological application.

## Results and discussion

### Isolation and identification

**Figure 1:**
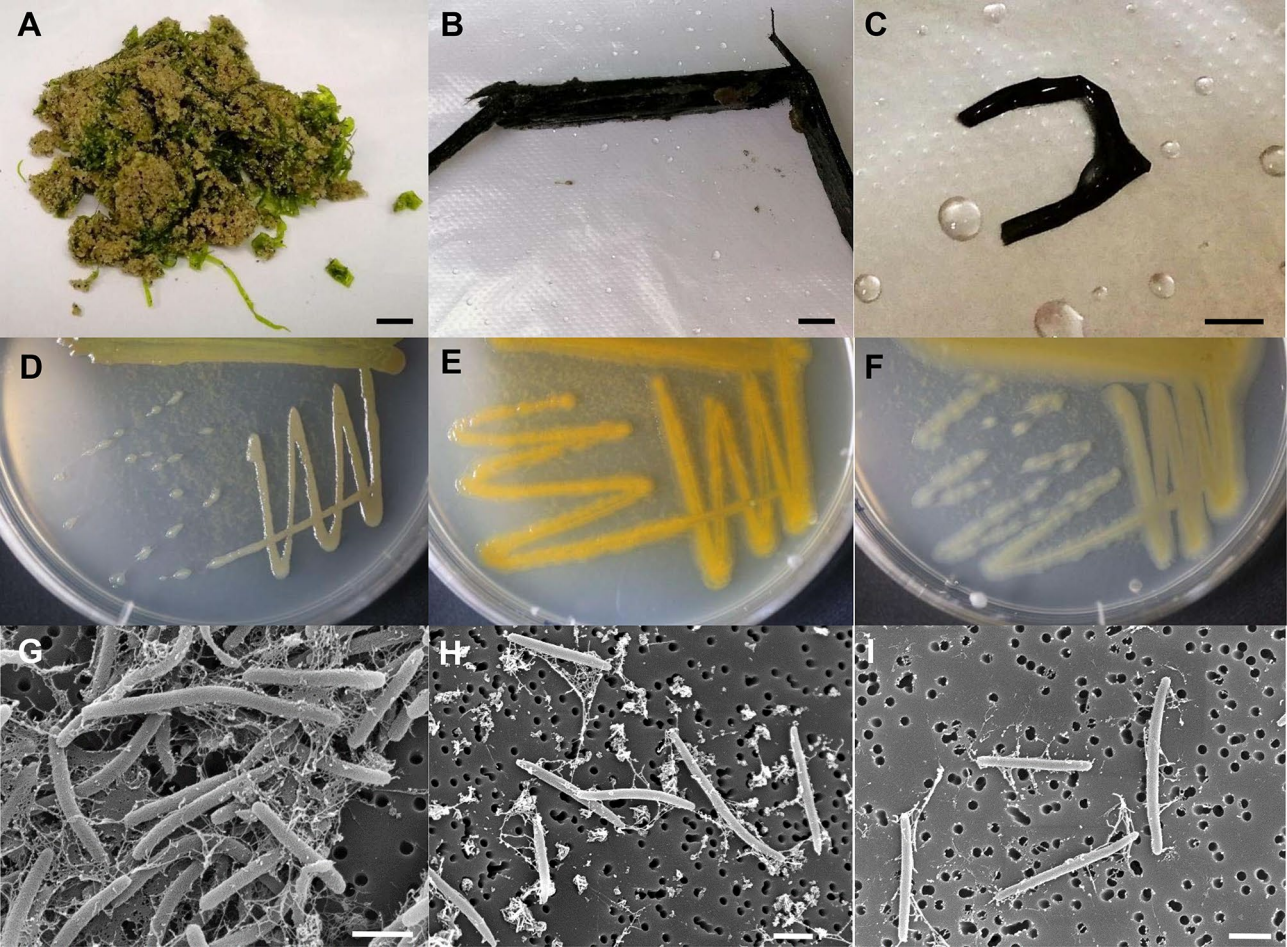
Origin, colony morphology, and cell morphology of three novel isolates in genus *Fulvivirga.* (**A**, **D**, **G**): strain SS9-22^T^; (**B**, **E**, **H**) strain W9P-11^T^; (**C**, **F**, **I**) strain SW1-E11^T^. A: seaweed collected at East Sea; B: degraded wood collected at Yellow Sea; C: seaweed collected at Yellow Sea; D, E, F: colony morphology of strains on MA plate; G, H, I: SEM images of cells of novel strains. Scale bar: 1 cm (**A,B**), 0.5 cm (**C**), 1 μm (**G,H,I**).

Strain SS9-22^T^ was isolated from a green alga *Ulva* sp. collected at the East Sea (Fig. 1A), and strains W9P-11^T^ and SW1-E11^T^ were isolated from a brown alga and decaying wood, respectively collected at the West Sea (Fig. 1B, C), the Republic of Korea, respectively. Pure cultures of the three isolates were obtained by selection of the gliding motility on a modified VY/2 medium (per liter, baker’s yeast, 5.0 g; CaCl_2_·2H_2_O, 1.0 g; vitamin B12, 0.5 mg, agar, 15 g) prepared to contain 60% strength seawater, buffered by HEPES (0.6 g/L) pH 7.2, and the three purified strains grew well on the marine agar (MA) (Fig. 1D, E, F). All the strains had irregular colonies on the solid medium. The color was brownish yellow for SS9-22^T^, orange for W9P-11^T^, and pale yellow for strain SW1-E11^T^ (Table 1). Cells of the three strains were rod-shaped with a length 2–5 µm and width 0.25–3.0 µm (Fig. 1G, H, I, and Table 1).

**Table 1:** Comparative physiological characteristics of three novel strains SS9-22^T^, W9P-11^T^, SW1-E11^T^ and their reference strains in genus *Fulvivirga*.

Characteristics	1	2	3	4	5	6	7	8	9	10
Cell shape	Rod	Rod	Rod	Rod	Rod	Long, slender, flexible rods	Rod	Rod	Rod	Rod
Colony morphology	Irregular, shiny, a clump on the central area, yellow to brownish	Irregular, shiny, orange	Irregular, pale yellow, smooth, a dark yellow in the central part of the colony	Irregular, pale yellow, dry surface	Circular, 1–2 mm in diameter, smooth, yellowish in colour, translucent and raised	Circular, smooth, mucoid, deep orange, glistening	2–3 mm in diameter, irregular, shiny, and yellow-brownish- coloured	Golden-yellow, circular, convex and smooth	Irregular, smooth, yellow to brownish	Golden-yellow, circular, convex and smooth
Width and length (μm)	0.29 and 2–3	0.3 and 2.8–5.0	0.25 and 2.7–4.1	0.2–0.3 and 4.0–6.0	0.3–0.5 and 5–6	0.5–0.7 and > 20	0.2–0.3 and 2.3–2.5	0.3 and 3.5–7.3	0.2–0.3 and 4.0–6.0	0.3–0.5 and 1–4
Gliding motility	+	+	+	+	−	+	+	+	−	+
Flexirubin-type pigment	−	+	+	−	−	−	−	−	−	−
NaCl tolerance (optimal) (% w/v)	0.5–15 (2)	0.5–12 (1–2)	0.5–12 (2)	0–9 (0.5)	2–12 (2–6)	0.5–12 (2–3)	0–10 (2–3)	0.5–6 (1)	0.5–7 (2–3)	0–6 (1–2)
Range of temperature (optimal) (℃)	10–45 (37)	10–37 (30)	10–37 (30)	16–48 (33)	30–37	15–45 (30–37)	14–44 (35–37)	25–37 (33)	10–33 (30)	20–42 (37)
Range of pH (optimal)	6.0–8.0 (7.0)	5.5–8.0 (6.0–7.0)	6.0–8.0 (7.0)	6.0–8.5 (7.5)	7.0–8.0	5.0–8.0 (7.0–8.0)	ND	5.5–8.5 (7.0)	5.5-~ (7.0–8.0)	6.0–8.0 (7.0)
Oxygen requirement	Aerobic	Aerobic	Facultative anaerobic	Facultative anaerobic	Aerobic	Strictly aerobic	Strictly aerobic	Facultative anaerobic	Strictly aerobic	Strictly aerobic

Taxa: 1, SS9-22^T^; 2, W9P-11^T^; 3, SW1-E11^T^; 4, *F. sediminis* 2943^T^ ^45^; 5, *F. imtechensis* JCM 17390^T^ ^41^; 6, *F. aurantia* KCTC 82638^T^ ^44^; 7, *F. kasyanovii* KCTC 12832^T^ ^40^; 8, *F. marina* 29W222^T^ ^45^; 9, *F. lutimaris* KCTC 42720^T^ ^43^; 10, *F. lutea* S481^T^ ^42^. ND: no determined.

To determine the taxonomic position of the isolates, the 16S rRNA gene sequences were determined. Alignment of the three 16S rRNA sequences on EzBioCloud website (https://www.ezbiocloud.net/) revealed that strain SS9-22^T^ was closest to strain *Fulvivirga kasyanovii* KCTC 12832^T^ with similarity of 98.1%; strains W9P-11^T^ and SW1-E11^T^ had the closest similarity to *F. sediminis* 2943^T^ of 94.9% and 99.8%, respectively (Table S1). The 16S rRNA sequences of SS9-22^T^, W9P-11^T^, and SW1-E11^T^ were registered as OM403091, OM403093, and OM403092 at GenBank, respectively.

**Figure 2:**
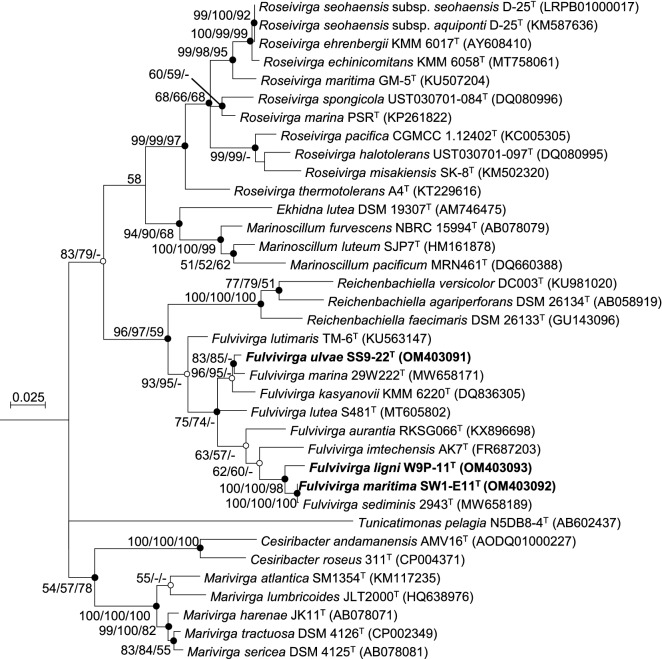
Maximum-likelihood phylogenetic tree constructed by MEGA7 software (version 7.0.26) based on 16S rRNA sequences showing the positions of three novel strains SS9-22^T^, W9P-11^T^, and SW1-E11^T^ with their closest representatives belonging to the order *Cytophagales.* Strain *Flavobacterium aquatile* NBRC 15052^T^ (GenBank accession number AB517711) was used as the outgroup. GenBank accession numbers are shown in parentheses. The 16S rRNA sequences were aligned by ClustalW and the result was trimmed in BioEdit software (version 7.2.5). The bootstrap resampling method of 1000 replicates was applied to evaluate the phylogenetic tree. Bootstrap values > 50% are presented. The closed circles stand for consensus of recovered nodes by using three algorithms, ML, NJ, and MP, respectively. The open circles stand for consensus of recovered nodes found from two out of three algorithms. Bar, 0.025 substitutions per nucleotide position.

Phylogenetic analysis based on 16S rRNA gene sequences showed that all three isolates belonged to a monophyletic clade of the genus *Fulvivirga.* The clustering was supported by high bootstrap values of 93% and 95% in maximum-likelihood and neighbor-joining algorithms, respectively (Fig. 2). Interestingly, inside the clade of the genus *Fulvivirga,* strain SS9-22^T^ created a separate cluster with strain *F. marina* 29W222^T^; but two strains, SW1-E11^T^ and W9P-11^T^, created a monophyletic cluster with strain *F. sediminis* 2943^T^ that was separated from strain SS9-22^T^. In addition, the similarity values of 16S rRNA gene among the three isolates was under 98.1% (Table S1) and the similarity values between the isolates and the existing seven species were lower than 98.1%, except the similarity value of 99.8% between strain SW1-E11^T^ with *F. sediminis* 2943^T^. To determine the exact phylogenetic position of the three strains*,* polyphasic taxonomy and a genome analysis were performed.

### Physiological characteristics

All three isolates were Gram-staining-negative, rod-shaped, mesophilic bacteria, which are shared in common with the existing species in the genus *Fulvivirga.* On the other hand, two strains, W9P-11^T^ and SW1-E11^T^, were distinguished from the other species in the genus *Fulvivirga* by containing flexirubin-type pigment. The colony morphologies of the three novel strains were irregular but those of the other species were circular. The colony colors were also different from other species in the genus *Fulvivirga* (Table 1, Fig. 1, and Fig. S1). Strain SW1-E11^T^ grew slowly under anaerobic or microaerophilic conditions, which is similar to *F. sediminis* 2943^T^ ^45^ and *F. marina* 29W222^T^ ^45^, while the two other novel isolates and the remaining species exclusively grew under an aerobic condition^40–44^. Furthermore, the three isolates showed gliding motility, which differed from *F. lutimaris* KCTC 42720^T^ ^43^ and *F. imtechensis* JCM 17390^T^ ^41^. Interestingly, even though strain SW1-E11^T^ and *F. sediminis* 2943^T^ share a high similarity of 16S rRNA gene (99.8%), their phenotypic characteristics had several differences. First, the colony of strain SW1-E11^T^ had a smooth and shiny surface, while the colony of *F. sediminis* 2943^T^ has a rough and dry surface (Fig. S1). Second, strain SW1-E11^T^ contained flexirubin-type pigment but *F. sediminis* 2943^T^ does not (tested in this study). The detailed characteristics among the three isolates and the existing species in genus *Fulvivirga* are presented in Table 1.

### Biochemical characteristics

The biochemical features of the three novel strains SS9-22^T^, W9P-11^T^, and SW1-E11^T^ shared common characteristics, such as starch degradation, oxidase and catalase activity, and utilization of D-cellobiose, dextrin, gentiobiose, D-glucose, D-melibiose, D-raffinose, D-trehalose, and D-turanose with the existing species in the genus *Fulvivirga*. Interestingly, only strain W9P-11^T^ was positive for D-glucuronic acid utilization, and only strains SS9-22^T^ and W9P-11^T^ utilized L-serine. Furthermore, only strains SW1-E11^T^ and *F. lutimaris* KCTC 42720^T^ showed *N-*acetyl-β-glucosaminidase activity. All three novel strains could hydrolyze Tweens 20 and 40, in contrast to *F. aurantia* KCTC 82638^T^ and *F. lutimaris* KCTC 42720^T^. Casein degradation was found in strains SS9-22^T^ and W9P-11^T^, but not in strain SW1-E11^T^. Chitin degradation was exhibited in strains SS9-22^T^, SW1-E11^T^, *F. imtechensis* JCM 17390^T^, and *F. kasyanovii* KCTC 12832^T^, but not in strains W9P-11^T^, *F. aurantia* KCTC 82638^T^, and *F. lutimaris* KCTC 42720^T^. Strains SW1-E11^T^ and *F. sediminis* 2943^T^ had several differences in biochemical characteristics. Strain SW1-E11^T^ was positive for DNase, β-galactosidase, and β-glucosidase activities, and showed an ability to utilize N-acetyl-D-glucosamine, glycyl-L-proline, melibiose, pectin, D-raffinose, sodium butyrate, D-trehalose, D-turanose, and stachyose while *F. sediminis* 2943^T^ is not able to utilize all of them^45^. More detailed differences to distinguish the three novel strains from the other species are presented in Table 2.

**Table 2:** Differential biochemical characteristics of novel strains SS9-22^T^, W9P-11^T^, SW1-E11^T^ and other species in genus *Fulvivirga*.

Characteristics	1	2	3	4	5	6	7	8	9	10
Hydrolysis of										
Casein	+	+	−	+	−	+	+	+	−	+
Chitin	+	−	+	+	+	−	+	w	−	+
Tween 20	+	+	+	+	+	−	+	+	−	+
Tween 40	+	+	+	+	+	−	+	+	−	+
Tween 80	−	−	+	+	+	+	+	+	−	+
Enzyme activities										
Crystine arylamidase	+	−	+	+	+	+	+	−	−	+
DNase	+	+	+	−	−	+	+	−	+	−
Esterase lipase	+	−	+	+	+	+	+	−	+	+
Gelatinase	+	+	+	+	+	−	+	ND	−	−
*N*-Acetyl-β-glucosaminidase	−	−	+	−	−	−	−	+	+	−
Nitrate reduction to nitrites	−	−	−	−	+	−	−	−	+	−
Trypsin	+	−	+	+	+	−	−	−	−	−
α-Chymotrypsin	−	−	−	−	+	−	−	−	+	−
α-Galactosidase	−	+	−	−	−	−	−	−	−	−
α-Glucosidase	+	−	−	−	−	−	−	−	−	−
β-Galactosidase	−	+	+	−	−	−	−	+	−	−
β-Glucosidase	+	+	+	−	−	−	−	−	+	−
H_2_S production	+	+	+	+	−	−	+	−	−	−
Utilization										
D-Glucose	+	+	+	+	−	−	+	+	−	−
L-Arabinose	−	−	+	+	−	−	−	−	−	−
*N*-Acetyl-glucosamine	−	−	+	+	−	−	−	−	−	ND
1% Sodium lactate	+	−	+	−	−	+	−	−	−	−
3-Methyl glucose	+	−	−	−	−	+	−	−	−	+
Acetic acid	+	+	+	+	−	ND	−	+	−	−
Acetoacetic acid	+	−	+	+	+	+	+	−	−	−
Aztreonam	+	−	−	−	+	ND	−	−	−	−
Cellobiose	+	+	+	+	−	+	+	−	+	+

	1	2	3	4	5	6*	7	8*	9	10^¶^
D-Fructose	−	+	−	+	−	+	+	+	−	+
D-Galactose	−	+	+	+	−	−	−	−	−	−
D-Galacturonic acid	+	+	+	+	−	−	+	−	−	−
D-Glucuronic acid	−	+	−	−	−	−	−	−	−	ND
D-Mannose	+	+	+	+	−	+	+	−	−	−
D-Raffinose	+	+	+	−	−	+	+	+	−	+
D-Salicin	+	+	+	+	−	−	+	−	−	−
D-Trehalose	+	+	+	−	−	+	+	+	+	+
D-Turanose	+	+	+	−	−	+	+	−	−	+
Gelatin	+	+	+	+	−	−	+	−	−	+
Gentiobiose	+	+	+	+	−	+	+	+	+	+
Glycyl-L-proline	+	+	+	−	−	−	+	−	−	ND
L-Arginine	+	−	−	−	−	−	+	−	−	+
L-Aspartic acid	+	+	+	+	−	−	−	+	−	+
L-Fucose	+	−	−	−	+	+	+	+	−	−
L-Galactonic acid lactone	+	+	+	+	−	−	+	−	−	−
L-Glutamic acid	+	+	+	+	−	+	+	+	−	−
Lithium chloride	+	−	−	−	−	+	−	−	−	ND
L-Malic acid	−	−	+	+	−	−	−	−	−	+
L-Serine	+	+	−	−	−	−	−	−	−	−
N-Acetyl-D-galactosamine	+	−	+	+	−	−	+	−	−	+
N-Acetyl-D-glucosamine	−	+	+	−	−	+	+	+	−	ND
N-Acetyl-β-D-mannosamine	−	−	−	−	−	+	−	−	−	+
Maltose	+	+	+	+	−	+	+	−	−	+
Maltodextrin	+	+	+	+	−	+	+	−	−	ND
Melibiose	+	+	+	−	−	+	+	−	+	−
Methyl β-D-glucoside	+	+	+	+	−	+	+	−	−	+
Pectin	−	+	+	−	−	+	+	+	−	ND
Potassium tellurite	−	+	+	+	−	−	+	−	−	ND
Quinic acid	+	−	−	−	−	−	−	−	−	+
Sodium butyrate	−	−	+	−	−	+	−	−	−	ND
Stachyose	+	+	+	−	−	−	+	−	−	ND
Sucrose	+	+	−	−	−	+	+	−	+	+
Tetrazolium blue	+	+	−	+	−	+	−	−	−	ND
α-D-Glucose	+	+	+	+	−	+	+	+	−	−
α-D-Lactose	+	+	+	+	−	ND	+	+	−	+
α-keto-Glutaric acid	+	−	−	−	−	+	−	−	−	ND

Taxa: 1, SS9-22^T^; 2, W9P-11^T^; 3, SW1-E11^T^; 4, *F. sediminis* 2943^T^; 5, *F. imtechensis* JCM 17390^T^; 6, *F. aurantia* KCTC 82638^T^; 7, *F. kasyanovii* KCTC 12832^T^; 8, *F. marina* 29W222^T^; 9, *F. lutimaris* KCTC 42720^T^; 10, *F. lutea* S481^T^. All strains utilize the following (Biolog GEN III): D-cellobiose, dextrin, D-melibiose, D-glucose, D-raffinose, D-trehalose, D-turanose, gentiobiose. w: weak. ND: no data available. All strains were positive for starch degradation and oxidase and catalase activities. *Data obtained from^44^. ^¶^Data obtained from^42^.

### Chemotaxonomic analysis

The major fatty acids (> 5.0%) of the three novel isolates were iso-C_15:0_, iso-C_17:0_ 3-OH, C_16:1_ ω5c, summed feature 3 (C_16:1_ ω7c/C_16:1_ ω6c), and iso-C_15:0_ 3-OH. Notably, strain SS9-22^T^ contained 7.6% iso-C_15:1_ G, which is similar to *F. aurantia* KCTC 82638^T^, *F. marina* 29W222^T^, and *F. lutimaris* KCTC 42720^T^; this component was lower in strains W9P-11^T^ (2.4%), SW1-E11^T^ (2.8%), *F. sediminis* 2943^T^ (2.3%), and *F. imtechensis* JCM 17390^T^ (3.7%), respectively. In addition, the summed feature 3 was higher than 10% in strains W9P-11^T^ and SW1-E11^T^, and *F. sediminis* 2943^T^ than in strain SS9-22^T^ and the remaining reference strains (Table 3). Together with the phylogenetic tree topology (Fig. 2), the clade comprising two novel isolates W9P-11^T^ and SW1-E11^T^, and two recognized species *F. sediminis* 2943^T^ and *F. imtechensis* JCM 17390^T^ could be distinguished from the rest of the species by consisting of a different percentage of fatty acid components of summed feature 3 (C_16:1_ ω7c/C_16:1_ ω6c) and iso-C_15:1_ G (Table 3).

**Table 3:** Composition (%) of the cellular fatty acid of three novel strains SS9-22^T^, W9P-11^T^, SW1-E11^T^ and other species in genus *Fulvivirga*.

Fatty acid	1	2	3	4	5	6	7	8	9	10
Straight-chain										
C_16:0_	1.4	1.4	1.2	1.5	–	1.4	1.5	–	1.2	4.4
C_14:0_	–	–	–	–	–	–	–	–	–	1.7
Unsaturated										
**C**_**16:1**_** ω5c**	**12.9**	**14.9**	**12.0**	**16.8**	**13.7**	**11.6**	**10.0**	**8.5**	**11.8**	**6.0**
Branched										
iso-C_13:0_	–	–	–	–	–	–	1.3	–	–	–
iso-C_14:0_	–	–	–	–	–	–	–	1	–	–
iso-C_15:1_ G	7.6	2.4	2.8	2.3	3.7	8.8	**14.4**	5.4	7.4	15.2
**iso-C**_**15:0**_	**30.4**	**31.6**	**38.1**	**35.0**	**36.3**	**34.9**	**21.5**	**32.6**	**33.3**	**36.3**
anteiso-C_15:0_	3.5	2.5	1.7	1.7	3.4	1.5	2.3	3.8	3.9	2.6
iso-C_16:0_	–	1.4	–	1.0	–	2.1	1.1	2.9	1.5	–
iso-C_17:0_	–	–	–	–	–	1.2	–	–	–	–
Hydroxy										
iso-C_15:0_ 3-OH	5.1	5.8	5.4	6.2	5.6	4.1	8.2	6.4	4.4	6.2
C_15:0_ 3-OH	–	1.7	1.3	–	–	–	2.2	–	–	2.6
iso-C_16:0_ 3-OH	1.0	–	–	–	–	–	2.7	1.5	–	1.5
C_16:0_ 3-OH	1.9	1.8	2.0	1.8	1.1	1.1	3.9	–	1.6	1.9
**iso-C**_**17:0**_** 3-OH**	**17.4**	**16.9**	**15.9**	**17.3**	**17.3**	**17.1**	**14.2**	**18.3**	**15.8**	**13.0**
C_17:0_ 3-OH	–	–	1.3	–	–	–	2.2	–	–	–
Summed features										
3	7.3	**11.4**	**13.0**	**11.6**	9.5	7.5	6.8	9.3	8.7	2.1
4	2.2	–	–	1.2	3.1	2.8	–	3.0	2.2	–
Others	9.3	8.2	5.3	3.6	6.3	5.9	7.7	7.3	8.2	6.5

Taxa: 1, SS9-22^T^; 2, W9P-11^T^; 3, SW1-E11^T^; 4, *F. sediminis* 2943^T^; 5, *F. imtechensis* JCM 17390^T^; 6, *F. aurantia* KCTC 82638^T^; 7, *F. kasyanovii* KCTC 12832^T^; 8, *F. marina* 29W222^T^; 9, *F. lutimaris* KCTC 42720^T^; 10, *F. lutea* S481^T^. Numbers indicate the percentages of the fatty acids. -, not detected (< 1%). Summed features 3: C_16:1_ ω7c/C_16:1_ ω6c; Summed features 4: C_17:1_ iso I/anteiso B. All data are from this study.

Values > 10% are in bold.

The polar lipid profiles of the three isolates were similar to that of the validly published species of the genus *Fulvivirga.* Strain SS9-22^T^ contained three aminophospholipids, four unidentified lipids, one unidentified aminolipid, one unidentified phospholipid, and one unidentified glycolipid. Strain W9P-11^T^ contained phosphatidylethanolamine (PE), three unidentified lipids, five unidentified aminolipids, two unidentified phospholipids, and three unidentified aminophospholipids. Meanwhile, strain SW1-E11^T^ contained phosphatidylethanolamine, three aminophospholipids, four unidentified lipids, one unidentified aminolipid, and one unidentified phospholipid. Interestingly, *F. sediminis* 2943^T^ does not contain any phospholipid in the polar lipid profile^45^, which differed from the novel strain SW1-E11^T^ (Fig. S2).

### Genome sequencing

The complete genomes of strains SS9-22^T^, W9P-11^T^, and SW1-E11^T^ were determined by a combination of Nanopore and Illumina sequencing platforms. Each of the three strains contained a single circular chromosome having size of 6.98, 6.52, and 6.39 Mb, respectively. The G + C content of the novel strains was from 38.1% to 41.9%, similar to the range of existing species of 37.3% to 42.7% (Table 4). CheckM analysis showed that the three assembled genomes have high completeness and low contamination (Table 4), which indicated the high quality and reliability of the genomes assembled by the combination of two sequencing methods. A comparison of genomic properties of the three isolates with known members in the genus *Fulvivirga* is presented in Table 4. Because all assembled genomes in the genus *Fulvivirga* have high completeness (> 98%), we could carry out detailed genomic analyses and comparisons.

**Table 4:** Comparative genome properties of three novel strains with existing members in genus *Fulvivirga*.

Feature	1	2	3	4	5	6	7	8	9	10
Accession no	CP089981	CP089979	CP089980	JAESIY-000000000	AMZN-00000000	SMMD-00000000	SMLW-00000000	JAEUGD-000000000	SMLV-00000000	CP070608
Approximate genome size (bp)	6,978,354	6,523,894	6,392,613	5,686,206	6,737,864	4,439,317	7,174,826	6,786,237	4,858,936	4,137,993
G + C content (%)	41.9	39.0	38.1	37.7	42.4	39.5	42.7	39.9	37.9	37.3
Completeness	100.0	99.9	99.9	100.0	100.0	98.2	98.3	100.0	99.1	99.9
Contamination	1.5	0.9	2.1	1.2	0.8	0.7	2.7	1.7	0.3	1.0
Genes total number	5686	5543	5354	4688	5661	4003	5958	5443	4209	3669
rRNAs (5S, 16S, 23S)	6	6	12	5	2	3	3	3	3	6
tRNAs	45	41	52	47	46	32	34	47	30	37
Genes assigned to COGs	1852	1774	1761	1673	1810	1460	1835	1758	1567	1529

Taxa: 1, SS9-22^T^; 2, W9P-11^T^; 3, SW1-E11^T^; 4, *F. sediminis* 2943^T^; 5, *F. imtechensis* JCM 17390^T^; 6, *F. aurantia* KCTC 82638^T^; 7, *F. kasyanovii* KCTC 12832^T^; 8, *F. marina* 29W222^T^; 9, *F. lutimaris* KCTC 42720^T^; 10, *F. lutea* S481^T^. COG: Clusters of Orthologous Groups of proteins.

**Figure 3:**
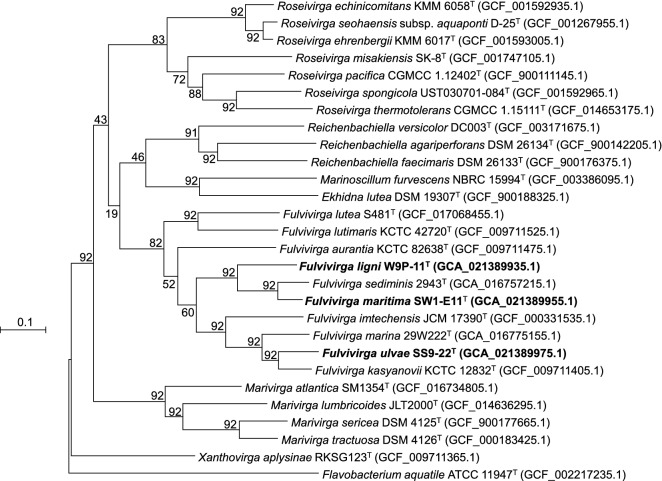
Maximum-likelihood phylogenetic tree showing relationship among SS9-22^T^, W9P-11^T^, and SW1-E11^T^ closely related species based on 92 core genes identified using the UBCG pipeline. GenBank accession numbers of the whole genome sequences are given in parentheses. *Flavobacterium aquatile* ATCC 11947^T^ (GCF_002217235) as the outgroup. Bootstrap values based on 1000 replicates are indicated at the branch nodes. Bar, 0.1 substitutions per site.

To check whether the genomes of the isolates are taxonomically different, average nucleotide identity (ANI) and digital DNA-DNA hybridization (dDDH) values were calculated. The ANI and the dDDH values among the three isolates and the existing species in the genus *Fulvivirga* (Table 5) were in ranges from 69.1% to 85.4% and 17.1% to 29.7%, respectively, which were significantly lower than the cut-off values of 95–96% for ANI value^46^ and 70% for dDDH value^47^ to distinguish bacterial species. Interestingly, although the 16S rRNA gene similarity of strain SW1-E11^T^ and *F. sediminis* 2943^T^ was 99.8%, the ANI and dDDH values were 84.34% and 27.4%, respectively, which were under the cut-off values to distinguish two species. The genome-based phylogenetic tree (Fig. 3) consistently exhibited not only the phylogenetic position of the three novel strains SS9-22^T^, W9P-11^T^, and SW1-E11^T^ inside the cluster of the genus *Fulvivirga*, as in the 16S rRNA-based phylogenetic tree (Fig. 2), but also separation of the isolates from the existing species, as the lower ANI and dDDH values showed. Hence, differentiation based on a whole genome analysis revealed that the three isolates represent three novel species in the genus *Fulvivirga.*

**Table 5:** ANI values calculated using EzBioCloud service and digital DNA-DNA hybridization values calculated on Genome-to-Genome Distance Calculator 3.0 among novel strains: 1, SS9-22^T^ (CP089981); 2, W9P-11^T^ (CP089979); 3, SW1-E11^T^ (CP089980), with the closest reference strains: 4, *F. sediminis* 2943^T^ (JAESIY000000000); 5, *F. imtechensis* JCM 17390^T^ (AMZN00000000); 6, *F. aurantia* KCTC 82638^T^ (SMMD00000000); 7, *F. kasyanovii* KCTC 12832^T^ (SMLW00000000); 8, *F. marina* 29W222^T^ (JAEUGD000000000); 9, *F. lutimaris* KCTC 42720^T^ (SMLV00000000); 10, *F. lutea* S481^T^ (CP070608).

Strain	1	2	3	4	5	6	7	8	9	10
ANI value (%)										
1	100.0	69.5	69.5	69.8	71.8	69.1	85.4	79.7	68.9	68.9
2	69.5	100.0	73.8	73.6	69.8	69.4	69.6	69.8	69.1	69.3
3	69.5	73.8	100.0	84.3	69.6	69.3	69.6	70.0	69.1	69.1
DNA-DNA hybridization (%)										
1	100.0	17.1	17.8	17.7	17.9	18.2	29.7	22.5	18.2	18.3
2	17.1	100.0	18.9	18.4	17.3	18.3	17.3	17.0	18	17.7
3	17.8	18.9	100.0	27.4	18.4	19.1	18.5	17.6	18.6	18.7

### Function annotation

Genome analysis revealed that three strains contain a number of genes to produce bioactive compounds and a large number of carbohydrate-active enzymes. antiSMASH^48^ analysis predicted several genes encoding polyketides and non-ribosomal peptide synthetase in the genome of strains SS9-22^T^, W9P-11^T^, and SW1-E11^T^. Almost all of the members of the genus *Fulvivirga* are anticipated to produce a high number of bioactive secondary metabolites (15–26 biosynthetic gene clusters (BGCs)), except *F. aurantia* KCTC 82638^T^, *F. marina* 29W222^T^, and *F. lutea* S481^T^ (2–4 BGCs) (Table S2). In all three isolates, a high number of genes were distributed into the cluster of orthologous groups (COGs) for amino acid transport and metabolism, followed by translation, ribosomal structure and biogenesis, and cell wall/membrane/envelope biogenesis (Fig. S3). Genes involved in carbohydrate utilization were identified based on the CAZy database (http://www.cazy.org/)^49^, which provides information of glycoside hydrolases (GHs, hydrolyze glycosidic bonds), glycosyltransferases (GTs, form glycosidic bonds), polysaccharide lyases (PLs, cleave glycosidic bonds through an eliminase mechanism), carbohydrate esterases (CEs, hydrolyze ester bonds), and auxiliary activities (AAs, redox enzymes acting with other CAZymes). The genome of strain SS9-22^T^ contained 325 CAZy modules in total, in which 112 genes encoded carbohydrate-degraded proteins. The genome of strain W9P-11^T^ contained a total of 354 CAZy modules, in which 187 annotated genes were related to carbohydrate degradation, i.e., GHs, PLs, and CEs. Meanwhile, the genome of strain SW1-E11^T^ encoded a total 260 CAZy modules, in which 138 genes encoded proteins related to GHs, PLs, and CEs (Table 6). The complete genome of *F. lutea* S481^T^ has been determined*,* and therefore we compared the genome of *F. lutea* S481^T^ with those of the three isolates to access carbohydrate degradation abilities by using CAZy database. Indeed, the number of CAZy modules of *F. lutea* S481^T^ is one-third that of the three isolates. Through the dbCAN server^50^, we could count the number of CAZy modules from the incomplete genomes of the other members in the genus *Fulvivirga* (Table S3). The GHs number of the three novel isolates was slightly lower than the number of genes annotated in the CAZy database. The genome of the three novel strains encoded a significantly higher number of GHs than those of *F. aurantia* KCTC 82638^T^, *F. imtechensis* JCM 17390^T^, *F. kasyanovii* KCTC 12832^T^, *F. lutea* S481^T^, and *F. lutimaris* KCTC 42720^T^ (Table S3). The genomes of strains W9P-11^T^ and SW1-E11^T^ had higher frequency of GHs (24.5 and 17.4 GHs per Mb, respectively) in comparison with the other species in the genus *Fulvivirga* (Table S3), except of *F. sediminis* 2943^T^ (21.79 GHs per Mb), and also higher than the median frequency of GHs (12 GHs per Mb) in the marine *Bacteroidota*^51^. Interestingly, the presence of CAZymes encoded in the genomes of the three novel strains was 1.4- to 3.5-fold higher than in the genomes of the other members belonging to the class *Flavobacteriia* (*Formosa agariphila* KMM 3901^T^, 193^37^; *Gramella flava* JLT2011, 184^51^; and *Polaribacter* spp., 100–146^38,52^), which are known as polysaccharide degraders.

**Table 6:** The carbohydrate-active enzyme (from CAZy database) compositions of three novel strains SS9-22^T^, W9P-11^T^, SW1-E11^T^, and a reference strain *F. lutea* S481^T^.

CAZyme	SS9-22^T^		W9P-11^T^		SW1-E11^T^		S481^T^	
	No. of genes	No. families	No. of genes	No. of families	No. of genes	No. of families	No. of genes	No. families
GH	86	38	165	54	114	37	25	11
GT	78	12	64	10	73	12	55	13
PL	12	5	8	5	12	5	–	–
CE	14	8	14	7	12	8	3	3
AA	1	1	–	–	1	1	–	–
CBM	48	15	103	17	48	8	17	4
Total	239	79	354	93	260	71	100	31

GH: glycoside hydrolase, GT: glycosyltransferase, PL: polysaccharide lyase, CE: carbohydrate esterase, AA: auxiliary activities, CBM: carbohydrate-binding modules.

The genes for polysaccharide degradation were identified through the servers of dbCAN^50^ and PULDB^53^ on CAZy^49^. Through the dbCAN server, the CAZyme gene clusters (CGCs)^54^, which have a similar arrangement of genes as in PUL, were found in all members in the genus *Fulvivirga* (Table S3). The genomes of strains SS9-22^T^, W9P-11^T^, and SW1-E11^T^ contained 58–70 CGCs, which were twofold greater than those of *F. aurantia* KCTC 82638^T^, *F. lutea* S481^T^, *F. lutimaris* KCTC 42720^T^; the number of CGCs encoded in the genome of strain SS9-22^T^ (58 CGCs) was similar to the numbers in *F. imtechensis* JCM 17390^T^ and *F. kasyanovii* KCTC 12832^T^ (Table S3). Through PULDB, the polysaccharide utilization loci (PULs) were found from the complete sequences of strains SS9-22^T^, W9P-11^T^, SW1-E11^T^, and *F. lutea* S481^T^; the PUL numbers were 24, 41, 32, and 4, respectively (Table S3). The distribution of CAZymes in PULs was different among the novel isolates and strain *F. lutea* S481^T^. Indeed, strain *F. lutea* S481^T^ has only four putative proteins related to carbohydrate degradation in PULs, while these numbers were 54, 143, and 47 in PULs of strains SS9-22^T^, W9P-11^T^, and SW1-E11^T^ genomes, respectively. This showed that the novel isolates may have higher potential for degradation of polysaccharides than known members of the genus *Fulvivirga*. Interestingly, the PULs of strain W9P-11^T^ contained a high number of carbohydrate-binding modules (CBMs) distributed in thirteen PULs. CBMs promote catalytic activity of the CAZyme by supporting the enzyme to bind to the target substrate, particularly insoluble polysaccharides, thus decreasing the distance between the enzyme and substrate^55^. The presence of a high number of CBMs indicates that strain W9P-11^T^ might effectively degrade the insoluble polysaccharide in the marine environment. Furthermore, the presence of several sulfatases (two genes from SS9-22^T^; one gene from strain W9P-11^T^) in the PULs of the three novel strains indicates that those PULs might degrade the sulfated polysaccharides. Through the PULDB, the putative PUL substrates could be predicted. In the genome of strain W9P-11^T^, PUL 10 harbored the double tandem gene *susC/susD* consecutive with the interleaved presence of five GH43 and two GH51, which were predicted to hydrolyze arabinan^56^. In addition, in the genome of strain SS9-22^T^, PUL 21 encoded the tandem *susC/susD* genes close to two GH16 and GH3. This was similar to PUL 139 and 142 of *Gillisia* spp. Hel1_29, Hel1_33_143, and PUL 173 of *Gramella* sp. MAR_2010_147 predicted to utilize laminarin^52^. Intriguingly, the strain SS9-22^T^ produced active laminarin-degrading enzymes in a broth culture (Table 7). Meanwhile, PUL 18 and PUL 23 of strain W9P-11^T^ contained abundant GH43, GH2, and GH92, which were predicted to hydrolyze mannose-rich substrates, similar to PUL 340 of *Salegentibacter* sp. Hel1_6^52^. Moreover, the abundance of CBM6, CBM13, CBM32, and CBM88 in those PULs indicated improvement of the catalytic activity by providing closer contact of GH enzymes to substrates^55^. Similar to strain W9P-11^T^, in the genome of strain SW1-E11^T^, PUL 2 also contained tandem *susC/susD* genes close to two GH51 and three GH43, which were predicted to degrade arabinan^56^. PUL 18 of strains SS9-22^T^, PUL 20, and PUL 38 of strain W9P-11^T^, PUL 23 of strain SW1-E11^T^, and PUL 2 of strain *F. lutea* S481^T^ contained GH13, and GH13 is predicted to be involved in hydrolysis of starch^29^. The presence of the PULs of starch utilization was consistent with the observation that the three novel strains and the existing type strains all showed starch degradation activity in vitro.

**Table 7:** Polysaccharide-degrading genes and in vitro activities of strains SS9-22^T^, W9P-11^T^, and SW1-E11^T^.

Metabolic substrate	SS9-22^T^		W9P-11^T^		SW1-E11^T^	
	in silico	in vitro	in silico	in vitro	in silico	in vitro
Alginate	PL6 (1), PL7 (3)	+	PL36 (1)	−	−	−
κ-Carrageenan	GH16 (3), sulfatase (2)	−	GH16 (5), sulfatase (1)	−	GH16 (3)	−
Cellulose	GH5 (4), GH9 (1)	−	GH5 (10), GH9 (1)	−	GH5 (8), GH9 (1)	−
Chitin	GH3 (7), GH5 (4), GH18 (4), GH20 (2), GH23 (5), GH48 (1)	−	GH3 (11), GH5 (10), GH18 (4), GH20 (2), GH23 (3), GH48 (1)	+	GH3 (11), GH5 (8), GH18 (4), GH20 (1), GH23 (4), GH48 (1)	−
Fucoidan	GH29 (1), GH95 (2), sulfatase (2)	−	GH29 (2), GH95 (2), sulfatase (1)	−	GH29 (2), GH95 (1)	−
Laminarin	PUL 21, GH16 (3)	+	GH16 (5)	−	GH16 (3)	+
Starch	GH13 (11), GH57 (2)	+	GH13 (8), GH57 (1)	+	GH13 (5), GH57 (1)	+
Xylan	GH3 (7), GH5 (4)	+	GH3 (11), GH5 (10), GH10 (4), GH30 (3)	+	GH3 (11), GH5 (8), GH10 (2), GH30 (3)	+

### Polysaccharide-degrading enzyme activity

The extracellular enzyme activities for degradation of alginate, κ-carrageenan, cellulose, chitin, fucoidan, laminarin, starch, and xylan were tested by detecting a reduced sugar by the 3, 5-dinitrosalicylic acid assay. All three strains SS9-22^T^, W9P-11^T^, and SW1-E11^T^ could degrade starch and xylan (Table 7). Degradation of starch was supported by the finding that all three strains contain a high number of GH13, which is majorly responsible for α-amylase^57,58^, and GH57 (Table S4). Moreover, strains SS9-22^T^, W9P-11^T^, and SW1-E11^T^ also contained numerous genes of xylanase belonging to families GH3^59^, GH5^60^, GH10^60^, and GH30^60,61^ (Table S4). Interestingly, strain SS9-22^T^ could degrade laminarin, and the genome of the strain contained PUL 21, which is very similar to the laminarin-specific PUL of *Gramella forsetii* KT0803^T^ in terms of gene organization^62^. This indicated that investigation of the gene construction in PULs could predict the candidate substrate. Only strain SS9-22^T^ among the three isolates could degrade alginate, and only strain W9P-11^T^ among the three isolates could degrade chitin. The genome of strain SS9-22^T^ had one PL6 and three PL7, which are responsible for alginate degradation^26^. The genome of strain W9P-11^T^ contained eleven GH3, ten GH5, four GH18, two GH20, three GH23, and one GH48, which are all known to participate in chitin degradation^63,64^. Detection of the polysaccharide degradation activities and the presence of corresponding genes indicate that the three novel strains could produce polysaccharide-degrading enzymes.

From the combination of genome-based and experiment-based analyses for polysaccharide degradation, the members of the genus *Fulvivirga* showed the traits of adaptation and specialization in polysaccharide degradation by the contribution of CAZyme^37,65^. Indeed, the strains isolated from algae, decaying wood, and sediment, including *F. ulvae* SS9-22^T^, *F. maritima* SW1-E11^T^, *F. ligni* W9P-11^T^, *F. sediminis* 2943^T^, *F. marina* 29W222^T^, and *F. lutimaris* KCTC 42720^T^, contained a high number of CAZy modules, which corresponded to more than 2.60% of the total genes (Table S3). Strain *F. aurantia* KCTC 82638^T^ isolated from seawater meanwhile contained a low number of CAZy modules of 55 genes, which is only 1.37% genes of the total genes. Furthermore, an in vitro test in this study showed that strains SS9-22^T^ and SW1-E11^T^ were able to degrade alginate, chitin, laminarin, starch, and xylan, which are algae-associated polysaccharides, by the corresponding CAZymes (Table 7). Taken together, the results show that the members of the genus *Fulvivirga* have high capability to degrade marine polysaccharides, and in particular the three novel isolates showed strongly higher potential in this regard than the known species.

Through a polyphasic approach, this study presented the three novel species in the genus *Fulvivirga* of phylum *Bacteroidota* as rich sources of carbohydrate-active enzymes and also as potential polysaccharide degraders. By the isolation method of mimicking nature conditions, the type strains of the three novel species were purely isolated. Analysis of genomes and a polysaccharide degradation assay of the three novel species helped to uncover the potential bio-production of the three novel species, providing information and a strategy for further study of active enzymes hydrolyzing marine polysaccharides.

### Description of *Fulvivirga ulvae* sp. nov.

*Fulvivirga ulvae* (ul'vae. L. gen. n. *ulvae* of *Ulva*, the name of the seaweed species from which is isolated).

Cells are gram-negative, mesophilic, neutrophilic, and rod-shaped. They are strictly aerobic and catalase and oxidase positive. Colonies on MB agar are irregular and shiny, forming a clump in the central area, and yellow to brownish in color. Growth occurs at 10–45 °C (optimum, 37 °C), at pH 6.0–8.0 (optimum, pH 7.0), and with 0.5–15% NaCl (optimum, 2%). H_2_S is produced. Positive for hydrolysis casein, gelatin, Tweens 20, 40, and degradation of alginate, laminarin, starch, and xylan. Negative for flexirubin-type pigment. Negative for hydrolysis of Tween 80. The major fatty acid components are iso-C_15:0_, iso-C_17:0_ 3-OH, and C_16:1_ ω5c.

The type strain, SS9-22^T^ (= KCTC 82072^T^ = GDMCC 1.2804^T^), was isolated from the green alga *Ulva* sp. The genome contains one circular chromosome 6.98 Mb long. The G + C content is 41.85%, as calculated from whole-genome sequencing.

### Description of *Fulvivirga ligni* sp. nov.

*Fulvivirga ligni* (lig'ni. L. gen. n. *ligni*, of wood, referring to the source of isolation).

Cells are gram-negative, mesophilic, neutrophilic, and rod-shaped. They are strictly aerobic and catalase and oxidase positive. Colonies on MB agar are irregular, shiny, and orange. Growth occurs at 10–37 °C (optimum, 30 °C), at pH 5.5–8.0 (optimum, pH 6.0–7.0), and with 0.5–12% NaCl (optimum, 1–2%). H_2_S is produced. Positive for hydrolysis of casein, chitin, gelatin, Tweens 20 and 40, and degradation of starch and xylan. Positive for flexirubin-type pigment production. Negative for hydrolysis of Tween 80. The major fatty acid components are iso-C_15:0_, iso-C_17:0_ 3-OH, C_16:1_ ω7c/C_16:1_ ω6c, and C_16:1_ ω5c.

The type strain, W9P-11^T^ (= KCTC 72992^T^ = GDMCC 1.2803^T^), was isolated from a degraded wood. The genome contains one circular chromosome 6.52 Mb long. The G + C content is 38.95%, as calculated from whole-genome sequencing.

### Description of *Fulvivirga maritima* sp. nov.

*Fulvivirga maritima* (ma.ri'ti.ma. L. fem. adj. *maritima* of the marine environment, maritime, referring to the habitat of isolation).

Cells are gram-stain-negative, mesophilic, neutrophilic, and rod-shaped. They are positive for micro-aerophilic, catalase and oxidase activities. Colonies on MB agar are irregular, pale yellow, smooth, and dark yellow in the center of the colonies. Growth occurs at 10–37 °C (optimum, 30 °C), at pH 6.0–8.0 (optimum, pH 7.0) and with 0.5–12% NaCl (optimum, 2%). H_2_S is produced. Positive for hydrolysis of gelatin, and Tweens 20, 40 and 80, and degradation of laminarin, starch, and xylan. Negative for hydrolysis of casein. Positive for flexirubin-type pigment production. The major fatty acid compositions components are iso-C_15:0_, iso-C_17:0_ 3-OH, summed feature 3 (C_16:1_ ω7c/C_16:1_ ω6c), and C_16:1_ ω5c.

The type strain SW1-E11^T^ (= KCTC 72832^T^ = GDMCC 1.2802^T^) was isolated from a dark green seaweed. The genome contains one circular chromosome 6.39 Mb long. The G + C content is 38.14%, as calculated from whole-genome sequencing.

## Materials and methods

### Origin of bacterial strains

Seaweed and degraded wood were collected in the North Pacific Ocean in the area belonging to the Republic of Korea. The brown seaweed and degraded wood were collected on October 14th, 2019, at Dongho-ri, Hae-myeon, Gochang-gun, Jeollabuk province (West Sea) (35°31′01.6″ N, 126°28′57.4″ E). The green alga *Ulva* sp. was collected on January 15th, 2020, at Sodol port, Jumunjin, Gangwon province (East Sea) (37°54′16.9″ N, 128°49′48.2″ E). For the isolation method, the strategy of imitating the natural conditions of the bacteria was applied. Indeed, the isolation medium was prepared based on sixty percent strength seawater (collected at the sampling site), supplied with 1.5% (w/v) agar (BD), and injected with 50 mg/L filtrated sterilization cycloheximide (Aldrich Sigma) after autoclaving the medium. In addition, a piece of filter paper (1 cm^2^, Whatman No.2) was put on the surface of the isolation agar plate as the carrier for the sample. A piece of each sample was then placed on the surface of the filter paper and inoculated at 28 ℃ in an aerobic condition. Subsequently, the signal of gliding bacteria that appeared on the surface of agar was observed under a stereomicroscope (ZEISS Stemi 508), and the gliding bacterial cells were picked up by a sharp needle (inner diameter of 0.26 mm) and transferred to a nutrient medium of 60% strength seawater buffered VY/2 medium (in 1 L: 600 mL seawater, 5 g baker’s yeast (Aldrich Sigma), 15 g agar, 400 mL distilled water, pH 7.0 ± 0.2, adjusted by 1 M NaOH, 25 mg filtrated-sterilization vitamin B_12_). The 60% strength seawater buffered VY/2 medium supports gliding motility of the target bacteria^66^. In the nutrient medium, after three to five days of incubation time, the edge of gliding cells was picked up and the cells were transferred to the fresh medium of 60% seawater buffered VY/2 agar plate, until obtaining the pure culture. All of the pure cultures were preserved in 20% glycerol at -80 ℃ and a lyophilized ampoule at 4 ℃. Pure cultures of the three novel strains were deposited at Korean Collection for Type Cultures (KCTC) and Guangdong Microbial Culture Collection Center (GDMCC).

### Phylogenetic analysis based on 16S rRNA gene sequence

To identify the three novel isolates, their 16S rRNA genes were amplified based on four universal primers, 27F^67^, 518F^68^, 805R^69^, and 1492R^67^, and sequenced by the Sanger method. The complete sequences were assembled manually by using NTI vector software^70^. Pairwise sequence alignment of the sequences was performed on EzBioCloud (https://www.ezbiocloud.net/). BioEdit software (version 7.2.5)^71^ was used for ClustalW multiple alignments and trimming the results. The trimmed file was used to construct phylogenetic trees based on three algorithms in MEGA7 software^72^ consisting of neighbor-joining (NJ)^73^, maximum-likelihood (ML)^74^, and maximum-parsimony (MP)^75^. The optimal model for the MP tree was the Kimura 2-parameter model, and the rates and patterns were gamma distributed with invariant sites (G + I), while the model of Kimura two-parameter^76^ was used for NJ and tree-bisection-reconnection (TBR) was used for the ML algorithm. The pairwise alignment among the three novel strains was calculated on BioEdit software (version 7.2.5)^71^ after trimming.

### Physiological characteristics

The physiological characteristics of all three strains were determined. All the experiments were duplicated. Morphology of colonies was observed on marine agar (MA) plates after three days’ cultivation in aerobic conditions. Gram staining was performed according to the standard protocol^77^ and the results were observed under a light microscope (Nikon Eclipse 80i). Cell morphology was observed through scanning electron microscope (SEM, JEOL JSM 7600F)^78^. The growth temperature was determined in marine broth (MB), for 7 days at 4, 10, 15, 20, 25, 30, 37, 45, and 60 °C, as described by Goldberg et al.^44^. The pH range for growth was adjusted in MB to pH 4.0–8.0 by using buffer phosphate/HCl Na_2_HPO_4_ 0.1 M/NaH_2_PO_4_ 0.1 M^44^, and pH 9.0–10.0 by using buffer Na_2_CO_3_ 0.1 M/NaHCO_3_ 0.1 M^79^, both at 0.5 pH unit intervals. The cells of the three novel strains were cultured in the buffered MB sterilized by membrane Millex^®^ VV 0.1 µm and incubated at 30 °C following Goldberg et al*.*^44^. Referring to Jung et al.^43^, the saline tolerance was determined on the supplementing MB, which was monitored with various concentrations of NaCl (0, 0.5 and 1.0–16.0% (w/v) at increments of 1.0%) at 30 °C, pH 7.0^44^. To assess the oxygen requirement, the three novel strains were cultivated on MA plates and incubated under aerobic, microaerophilic (in a closed jar with a package of BD GasPak EZ CO_2_ container system), and anaerobic conditions (in a closed jar with a package of BD GasPak EZ anaerobe container system) for one week at 28 °C. To assess the flexirubin-type pigments, drops of 20% KOH solution were added to the surface of the colonies and the positive and negative results were monitored based on the changing color of the colonies, as described in^80^. Gliding activity was tested by the hanging drop method, as described by Bowman^81^.

### Biochemical characteristics

Cells of the three novel isolates and their reference strains cultured on MA at 30 °C for two days were used to identify the biochemical characteristics. The cells were parallel inoculated on API ZYM, API 20NE (bioMerieux), and GEN III MicroPlates (Biolog) according to the manufacturers’ instructions, except that the saline solution was included in the inoculating fluids for a final concentration of 2% (w/v)^44^. Hydrolysis of starch was tested on MA with supplied 0.2% (w/v) starch and detected by a clear zone after staining with iodine solution^82^. Hydrolysis of cellulose was assessed on a CMC agar plate (in 1 L: 1 g NH_4_H_2_PO_4_, 0.2 g KCl, 1 g MgSO_4_.7H_2_O, 1 g yeast extract, 26 g carboxymethylcellulose sodium salt, 20 g NaCl, 15 g agar, in 1 L of artificial seawater^83^), and detected by a clear zone after embedding in Congo Red and washing with 1% NaCl solution. Chitin-degrading activity was examined on a minimal salt medium (in 1 L: 0.5 g KH_2_PO_4_, 1.5 g K_2_HPO_4_, 1 g NH_4_NO_3_, 20 g NaCl, 1 mg yeast extract, 0.5 g chitin, pH 7.0, 20 g agar, distilled water 1000 mL) according to Xu et al*.*^84^ for seven days at 30 ℃. Hydrolysis of Tweens 20, 40, and 80 (1%, v/v) was determined by using MA as basal media^79,85^. H_2_S production was tested on MB, supplied with 5 g/L sodium thiosulfate, and detected by using a filter-paper strip impregnated with lead acetate^79,85^. In order to determine the catalase activity, 3% H_2_O_2_ solution was dropped on the surface of the cells^82^. Oxidase activity was tested by the reaction of the cells to oxidase reagent (bioMerieux). DNase activity was examined on DNase agar (Difco) using artificial seawater^83^ with 2% NaCl instead of distilled water. Gelatinase activity was tested on nutrient gelatin (Remel Gelatin medium), in which distilled water was replaced by artificial seawater^83^ with 2% NaCl, for one week at 25 ℃, and a positive result was recognized by the presence of liquid-stage medium^82^.

### Chemotaxonomic characteristics

Chemotaxonomic features of SS9-22^T^, W9P-11^T^, and SW1-E11^T^ were determined. For the fatty acid profile of the three novel isolates and their reference strains, cells cultivated on MA for two days were harvested. The standard MIDI protocol^86^ (version 6.2) was applied to extract the fatty acid components. The extracted fatty acids methyl esters were then injected into a gas chromatograph^86^, and the components were identified based on the TSBA 6.0 database^87^. The quinone types of SS9-22^T^, W9P-11^T^, and SW1-E11^T^ were extracted from 100 mg of freeze-dried cells of each strain by shaking in chloroform–methanol (2:1, v/v) overnight. The extracts were concentrated and redissolved in 100% acetone. The acetone suspension was applied to thin-layer chromatography (TLC, Kieselgel 60F_254_, 20 × 20 cm, Merck), and separated in a combined solvent of petroleum ether-diethyl ether (9:1, v/v). The band detected under UV light was marked, harvested, and recovered in acetone. The extracts were further purified via reversed-phase high-performance liquid chromatography (LC20AD system, Shimadzu) using an ODS-2 (C18) column (150 × 4.6 mm I.D; YMC HPLC column), with a combination of methanol-isopropyl ether (3:1, v/v) as the mobile phase and wavelength of 270 nm to detect the quinone components^87^. The polar lipids of the three novel strains were extracted from their freeze-dried cells according to the detailed method described by Komagata and Suzuki^88^. Extracted lipids were applied on a quarter of a silica gel TLC plate, and the first dimension was developed in combined solvents of chloroform–methanol-water (65:25:4, v/v/v), and subsequently the second dimension was developed in a solvent system of chloroform–methanol-acetic acid–water (80:15:12:4, v/v/v/v)^87^. The TLC plates were sprayed with various appropriate reagents to identify the polar lipid profiles of the novel isolates, including molybdatophosphoric acid to identify total lipids, ninhydrin for the amino groups, molybdenum blue for phosphate groups, and α-naphthol in a sulphuric acid solution to detect the sugar groups^89^.

### Genome analysis

Genomic DNA of strains SS9-22^T^, W9P-11^T^, and SW1-E11^T^ was extracted from a two-day culture on a MA plate by using a NucleoSpin Microbial DNA kit (MACHEREY–NAGEL, Germany), according to manufacturer’s instructions. The quality of genomic DNA was quantified by Nanodrop 2000/2000c and the size length was monitored on 1% agarose electrophoresis gel.

The whole-genome sequences of the three novel isolates were determined by the combination of two platform methods, the Illumina platform (at Macrogen, Inc., Seoul, Republic of Korea) and Nanopore platform (at Biological Resource Center, Korea Research Institute of Bioscience and Biotechnology, Republic of Korea). For Illumina sequencing, the short-length DNA was used to build up a library based on the protocol of TruSeq DNA PCR-Free sample preparation guide, part #15036187 Rev. D. For nanopore sequencing, the high-molecular-weight DNA was used to prepare the library according to the Native barcoding genomic DNA protocol (with EXP-NBD104, and SGK-LSK109, version NBE_9065_v109_revV_14Aug2019). The genomes were de novo assembled by Canu (version 2)^90^. Medaka (version 1.3.2, https://github.com/nanoporetech/medaka) was used as a polishing tool for assembly by counting the occurrences of each nucleotide at each position on the assembled sequence to predict the true base at that position. The quality of the assembled genomes and annotation completeness were quantified on BUSCO (https://busco.ezlab.org/)^91^. The contamination and the completeness of the genomes were estimated by CheckM (version 1.1.3)^92^. Genomes were annotated on Prokka (version 1.12)^93^. The digital DNA-DNA hybridization was calculated the average nucleotide identity (ANI) tool on EzBioCloud (https://www.ezbiocloud.net/tools/ani)^46^, and genome-to-genome distance calculator (version 2.1) on DSMZ (https://ggdc.dsmz.de/ggdc.php#)^47^. From the whole genome sequence, the G + C content was calculated. The gene sequences obtained from the Prokka pipeline were annotated with the COG database^94^ using RPS-BLAST^95^ (e-value = ${10}^{-4}$) integrated in WebMGA (https://github.com/weizhongli/webMGA)^96^. Carbohydrate-active enzymes were annotated using the dbCAN2 meta server^50^ and CAZy database^97^. Biosynthetic gene clusters (BGCs) were predicted by antiSMASH 6.0^48^.

The whole-genome-based phylogenetic tree was constructed on the up-to-date bacterial core gene (UBCG) pipeline containing 92 core genes^98^. *Flavobacterium aquatile* ATCC 11947^T^ (GCF_002217235) as the outgroup.

### Polysaccharide-degrading enzyme activity assay

To test the activity of polysaccharide-degrading enzymes, the liquid medium was prepared by adding the following polysaccharide substrates to the marine broth: κ-carrageenan, cellulose, chitin, sodium alginate, starch, and xylan 0.2% (w/v); fucoidan and laminarin 0.1% (w/v)^99^. The cells harvested on day two on MA plates were inoculated. The initial cell concentration was set the same at OD_600nm_ 0.2. The negative control was the culture medium without bacterial cells. After three days, the supernatant of the culture was harvested and reacted with 3, 5-dinitrosalicylic acid (DNS)^100^ to detect reducing sugar production. In brief, the supernatant was reacted with DNS reagent (1:3, v/v) in a glass test tube, and then the tube was heat in a boiled-water bath for 5 min. The tubes were cooled under tap water. The absorbance at 570 nm was measured to detect any reducing sugar released from the degradation of polysaccharides^100^.

## Supplementary Information

Supplementary Information.

## Data Availability

The datasets generated and analysed during the current study are available in the NCBI repository. GenBank accession number of 16S rRNA gene sequences of the strains SS9-22^T^, W9P-11^T^, and SW1-E11^T^ are OM403091, OM403093, and OM403092, respectively. GenBank sequence numbers are CP089981, CP089979, and CP089980, respectively.
