# Supplementary material for: Three marine species of the genus Fulvivirga, rich sources of carbohydrate-active enzymes degrading alginate, chitin, laminarin, starch, and xylan
Source: Sci Rep. 2023 Apr 18;13:6301. doi: 10.1038/s41598-023-33408-4 (PMC10113389; doi:10.1038/s41598-023-33408-4)
Supplement: Supplementary file 1 — Supplementary Information. [file 41598_2023_33408_MOESM1_ESM.docx]

**Supplementary Material**

**Three marine species of the genus *Fulvivirga,* rich sources of carbohydrate-active enzymes degrading alginate, chitin, laminarin, starch, and xylan.**

Tra T. H. Nguyen^1, 2^, Tien Q. Vuong^3^, Ho Le Han^1, 4^, Zhun Li^1, 2^, Yong-Jae Lee^1^, Jaeho Ko^1^, Olga I. Nedashkovskaya^5^, Song-Gun Kim^1, 2, *^

^1^Biological Resource Center, Korean Collection for Type Cultures, Korea Research Institute of Bioscience and Biotechnology, Jeongeup 56212, Republic of Korea.

^2^Department of Biotechnology, KRIBB School, University of Science and Technology (UST), Daejeon 34113, Republic of Korea.

^3^Hanoi University of Science, Vietnam National University, Hanoi 10000, Vietnam.

^4^Scientific Management Department, Dong A University, Danang 50000, Vietnam.

^5^G.B. Elyakov Pacific Institute of Bioorganic Chemistry of the Far-Eastern Branch of the Russian Academy of Sciences, Vladivostok 690022, Russia.

*Corresponding author: sgkim@kribb.re.kr.

**Table S1**. 16S rRNA gene sequence similarity among novel strains: 1, SS9-22^T^ (OM403091); 2, W9P-11^T^ (OM403093); 3, SW1-E11^T^ (OM403092), with the closest reference strains: 4, *F. sediminis* 2943^T^ (MW658189); 5, *F. imtechensis* JCM 17390^T^ (FR687203); 6, *F. aurantia* KCTC 82638^T^ (KX896698); 7, *F. kasyanovii* KCTC 12832^T^ (DQ836305); 8, *F. marina* 29W222^T^ (MW658171); 9, *F. lutimaris* KCTC 42720^T^ (KU563147); 10, *F. lutea* S481^T^ (MT605802).

| Strain | 1 | 2 | 3 | 4 | 5 | 6 | 7 | 8 | 9 | 10 |
| --- | --- | --- | --- | --- | --- | --- | --- | --- | --- | --- |
| SS9-22^T^ | 100 | 94.4 | 97.2 | 94.9 | 96.4 | 94.3 | 98.1 | 97.7 | 96.2 | 95.8 |
| W9P-11^T^ | 94.4 | 100 | 95.1 | 97.0 | 94.9 | 94.6 | 94.3 | 94.0 | 93.8 | 93.6 |
| SW1-E11^T^ | 97.2 | 95.1 | 100 | 99.8 | 95.6 | 94.9 | 94.5 | 93.8 | 93.8 | 93.7 |

**Table S2**. Comparison of biosynthetic gene clusters (BGCs) among three novel strains with existing members in genus *Fulvivirga* based on antiSMASH analysis*.* Taxa: 1, SS9-22^T^; 2, W9P-11^T^; 3, SW1-E11^T^; 4, *F. sediminis* 2943^T^; 5, *F. imtechensis* JCM 17390^T^; 6, *F. aurantia* KCTC 82638^T^; 7, *F. kasyanovii* KCTC 12832^T^; 8, *F. marina* 29W222^T^; 9, *F. lutimaris* KCTC 42720^T^; 10, *F. lutea* S481^T^.

| Type | **1** | **2** | **3** | **4** | **5** | **6** | **7** | **8** | **9** | **10** |
| --- | --- | --- | --- | --- | --- | --- | --- | --- | --- | --- |
| Arylpolyene | 1 | 2 | 2 | 1 | 1 | - | 1 | 1 | - | - |
| HgIE-KS | - | - | 1 | 1 | - | - | - | - | - | - |
| Lanthipeptide class I | 1 | 1 | 1 | 1 | 2 | - | 1 | 1 | - | - |
| Lanthipeptide class IV | - | - | - | 1 | - | - | - | - | - | - |
| NRPS | 3 | 1 | 2 | 5 | 12 | - | 8 | 10 | - | - |
| NRPS-like | 1 | 2 | 2 | 1 | 2 | 1 | 3 | 2 | - | - |
| Resorcinol | 1 | 1 | 1 | - | - | - | 1 | 1 | - | - |
| RiPP-like | - | 1 | - | - | - | - | - | 1 | - | - |
| Siderophore | 1 | 2 | 1 | 1 | - | - | 1 | 1 | - | - |
| T1PKS | 4 | 2 | 3 | 5 | 3 | 1 | 2 | 5 | - | - |
| T3PKS | 1 | 1 | 1 | 1 | 1 | 1 | 1 | 1 | 1 | 1 |
| Terpene | 1 | 1 | 1 | 2 | 1 | 1 | 1 | 1 | 1 | 1 |
| LAP | - | 1 | - | - | - | - | - | - | - | - |
| PKS-like | 1 | - | - | - | 1 | - | 1 | 1 | - | - |
| TransAT-PKS | 1 | - | - | - | 1 | - | 4 | 1 | - | - |
| Phosphonate | - | - | - | - | - | - | - | - | - | 1 |
| Total | 16 | 15 | 15 | 19 | 24 | 4 | 24 | 26 | 2 | 2 |

**Table S3.** Distribution of CAZyme in the genome of species in genus *Fulvivirga* through dbCAN server. GH: Glycoside hydrolase; PUL: polysaccharide utilization loci (through CAZy database).

| Strain name | Source of isolation | Genome size (Mbp) | Genes | dbCAN | | | | CAZyme  (% gene) | CAZy database |
| --- | --- | --- | --- | --- | --- | --- | --- | --- | --- |
|  |  |  |  | CAZy modules | GHs | GH/Mbp | CGCs |  | PUL |
| *F. ulvae* SS9-22^T^ | Algae | 6.98 | 5,686 | 164 | 76 | 10.89 | 58 | 2.89 | 24 |
| *F. ligni* W9P-11^T^ | Decaying wood | 6.52 | 5,543 | 214 | 160 | 24.54 | 66 | 3.86 | 32 |
| *F. maritima* SW1-E11^T^ | Algae | 6.39 | 5,354 | 264 | 111 | 17.37 | 70 | 4.93 | 41 |
| *F. sediminis* 2943^T^ | Sediment | 5.69 | 4,688 | 197 | 124 | 21.79 | 69 | 4.20 | - |
| *F. imtechensis* JCM 17390^T^ | Seawater | 6.74 | 5,661 | 135 | 54 | 8.01 | 50 | 2.39 | - |
| *F. aurantia* KCTC 82638^T^ | Sponge | 4.44 | 4,003 | 55 | 16 | 3.60 | 24 | 1.37 | - |
| *F. kasyanovii* KCTC 12832^T^ | Seawater | 7.17 | 5,958 | 171 | 82 | 11.44 | 51 | 2.87 | - |
| *F. marina* 29W222^T^ | Sediment | 6.79 | 5,443 | 171 | 63 | 9.28 | 60 | 3.14 | - |
| *F. lutimaris* KCTC 42720^T^ | Sediment | 4.86 | 4,209 | 113 | 58 | 11.93 | 38 | 2.69 | - |
| *F. lutea* S481^T^ | Seawater | 4.14 | 3,669 | 78 | 25 | 6.04 | 27 | 2.13 | 4 |

**Table S4**. CAZyme components encoded in genomes of three novel strains SW1-E11^T^, W9P-11^T^, and SS9-22^T^ through dbCAN server, number of appearances in parentheses. Note: AA: Auxiliary Activities, CE: Carbohydrate esterase, CBM: Carbohydrate-Binding Module, GH: Glycoside Hydrolase, GT: GlycosylTransferase, PL: Polysaccharide lyase.

| No. of family | SS9-22^T^ | W9P-11^T^ | SW1-E11^T^ |
| --- | --- | --- | --- |
| 1 | CBM13 | AA2 | AA10 |
| 2 | CBM32 | CBM13 (3) | AA2 |
| 3 | CBM35 | CBM32 | CBM13 (3) |
| 4 | CBM48 + GH13_9 | CBM35 (2) | CBM6 |
| 5 | CBM5 | CBM6 (6) | CBM88 |
| 6 | CBM6 | CBM9 | CBM9 |
| 7 | CBM9 | CE11 | CE1 (2) |
| 8 | CE11 | CE12 (2) | CE11 |
| 9 | CE12 (2) | CE15 | CE12 (2) |
| 10 | CE2 | CE4 (2) | CE4 (2) |
| 11 | CE4 | CE8 (2) | CE8 (2) |
| 12 | CE8 (4) | GH10 (4) | CE8 + CBM6 + PL1_2 + CBM88 + CBM35 |
| 13 | GH105 | GH105 | GH10 |
| 14 | GH109 | GH11 + CBM60 | GH10 + CBM64 |
| 15 | GH13 | GH115 | GH104 |
| 16 | GH13_10 + CBM48 (2) | GH125 | GH105 |
| 17 | GH13_16 | GH13 (3) | GH125 |
| 18 | GH13_26 | GH13_10 + CBM48 | GH127 |
| 19 | GH13_3 | GH13_31 | GH128 |
| 20 | GH13_31 | GH13_36 | GH13 |
| 21 | GH13_36 | GH13_7 | GH13_10 |
| 22 | GH13_7 | GH13_8 + CBM48 | GH13_36 |
| 23 | GH130 (2) | GH130 (4) | GH13_7 |
| 24 | GH133 | GH142 | GH13_8 |
| 25 | GH144 (2) | GH143 | GH130 (2) |
| 26 | GH15 | GH144 (2) | GH143 |
| 27 | GH16_3 (2) | GH146 (2) | GH144 |
| 28 | GH171 | GH147 | GH146 (2) |
| 29 | GH18 (2) | GH15 (2) | GH147 |
| 30 | GH18 + CBM6 | GH16 + CBM32 | GH15 (2) |
| 31 | GH18 + CBM73 | GH16 + CBM6 + CBM56 | GH16_3 |
| 32 | GH2 (3) | GH16_3 | GH171 |
| 33 | GH20 (2) | GH16_3 + CBM6 | GH18 + CBM5 |
| 34 | GH23 (3) | GH16_3 + GH128 + CBM6 + CBM32 | GH2 (9) |
| 35 | GH26 (2) | GH171 | GH20 |
| 36 | GH27 | GH18 | GH23 (2) |
| 37 | GH28 (2) | GH18 + CBM5 | GH23 + CBM50 (2) |
| 38 | GH29 | GH18 + CBM6 | GH25 |
| 39 | GH3 (7) | GH2 (14) | GH26 |
| 40 | GH31 (3) | GH2 + CBM32 (2) | GH26 + CBM35 |
| 41 | GH37 | GH2 + CBM57 | GH26 + GT2 |
| 42 | GH39 | GH20 (2) | GH27 |
| 43 | GH43_10 | GH23 | GH28 |
| 44 | GH43_9 | GH23 + CBM50 (3) | GH29 |
| 45 | GH48 | GH26 (4) | GH29 + CBM32 |
| 46 | GH5_26 | GH26 + CBM35 | GH3 (11) |
| 47 | GH5_4 (2) | GH27 | GH30_3 |
| 48 | GH5_7 | GH28 | GH30_4 |
| 49 | GH51 | GH29 | GH30_8 |
| 50 | GH57 (2) | GH29 + CBM32 | GH31 (7) |
| 51 | GH63 | GH3 (10) | GH35 |
| 52 | GH64 + CBM6 | GH3 + CBM32 | GH37 |
| 53 | GH65 | GH30_1 | GH39 |
| 54 | GH77 | GH30_3 (2) | GH43_10 |
| 55 | GH8 + CBM32 | GH31 (4) | GH43_24 |
| 56 | GH81 + CBM6 | GH32 (2) | GH43_28 (2) |
| 57 | GH9 + CBM64 +CBM3 | GH35 | GH43_29 |
| 58 | GH92 (5) | GH37 | GH43_34 |
| 59 | GH95 (2) | GH38 | GH43_34 + CBM32 |
| 60 | GH97 (2) | GH43 | GH43_37 + CBM61 |
| 61 | GT1 (2) | GH43_10 (2) | GH43_5 |
| 62 | GT19 | GH43_11 | GH43_5 + CBM13 |
| 63 | GT2 (24) | GH43_12 | GH43_9 |
| 64 | GT20 | GH43_26 (2) | GH44 + CBM3 |
| 65 | GT28 | GH43_28 + CBM32 (2) | GH48 |
| 66 | GT30 | GH43_29 | GH5 + CBM6 + CBM13 |
| 67 | GT4 (11) | GH43_32 + CBM6 | GH5_1 |
| 68 | GT5 | GH43_34 (3) | GH5_2 + CBM6 |
| 69 | GT51 (3) | GH43_4 | GH5_26 |
| 70 | GT83 (2) | GH43_5 | GH5_4 (3) |
| 71 | GT9 (2) | GH43_5 + CBM13 | GH5_46 |
| 72 | PL1 (3) | GH43_8 | GH5_7 |
| 73 | PL1_2 (2) | GH48 | GH51 (3) |
| 74 | PL6_1 | GH5 + CBM6 + CBM13 | GH53 |
| 75 | PL7_5 (2) | GH5_1 | GH64 + CBM6 |
| 76 |  | GH5_13 | GH67 |
| 77 |  | GH5_2 + CBM6 | GH73 |
| 78 |  | GH5_26 | GH74 |
| 79 |  | GH5_4 (2) | GH84 |
| 80 |  | GH5_46 + CBM6 | GH88 |
| 81 |  | GH5_7 (2) | GH9 |
| 82 |  | GH51 (3) | GH9 + CBM64 |
| 83 |  | GH53 | GH92 (4) |
| 84 |  | GH62 | GH95 |
| 85 |  | GH64 + CBM6 | GH97 (3) |
| 86 |  | GH65 | GT1 (3) |
| 87 |  | GH67 | GT19 |
| 88 |  | GH76 (4) | GT2 (22) |
| 89 |  | GH78 | GT20 |
| 90 |  | GH81 + CBM32 + CBM38 | GT28 |
| 91 |  | GH81 + CBM6 | GT3 |
| 92 |  | GH87 | GT30 |
| 93 |  | GH87 + CBM9 | GT4 (13) |
| 94 |  | GH88 (2) | GT5 |
| 95 |  | GH9 + CBM64 +CBM3 | GT51 (3) |
| 96 |  | GH92 (10) | GT9 |
| 97 |  | GH95 (2) | PL1_2 (2) |
| 98 |  | GH97 (5) | PL11_1 |
| 99 |  | GT1 | PL11_1 + CBM35 |
| 100 |  | GT19 (2) | PL42 |
| 101 |  | GT2 (18) | PL9 (2) |
| 102 |  | GT20 | PL9_1 |
| 103 |  | GT28 | PL9_1 + CBM35 (2) |
| 104 |  | GT3 |  |
| 105 |  | GT4 (14) |  |
| 106 |  | GT5 |  |
| 107 |  | GT51 (3) |  |
| 108 |  | GT83 (2) |  |
| 109 |  | GT9 |  |
| 110 |  | PL1_2 |  |
| 111 |  | PL29 |  |
| 112 |  | PL42 |  |
| 113 |  | PL9 |  |
| 114 |  | PL9_1 |  |


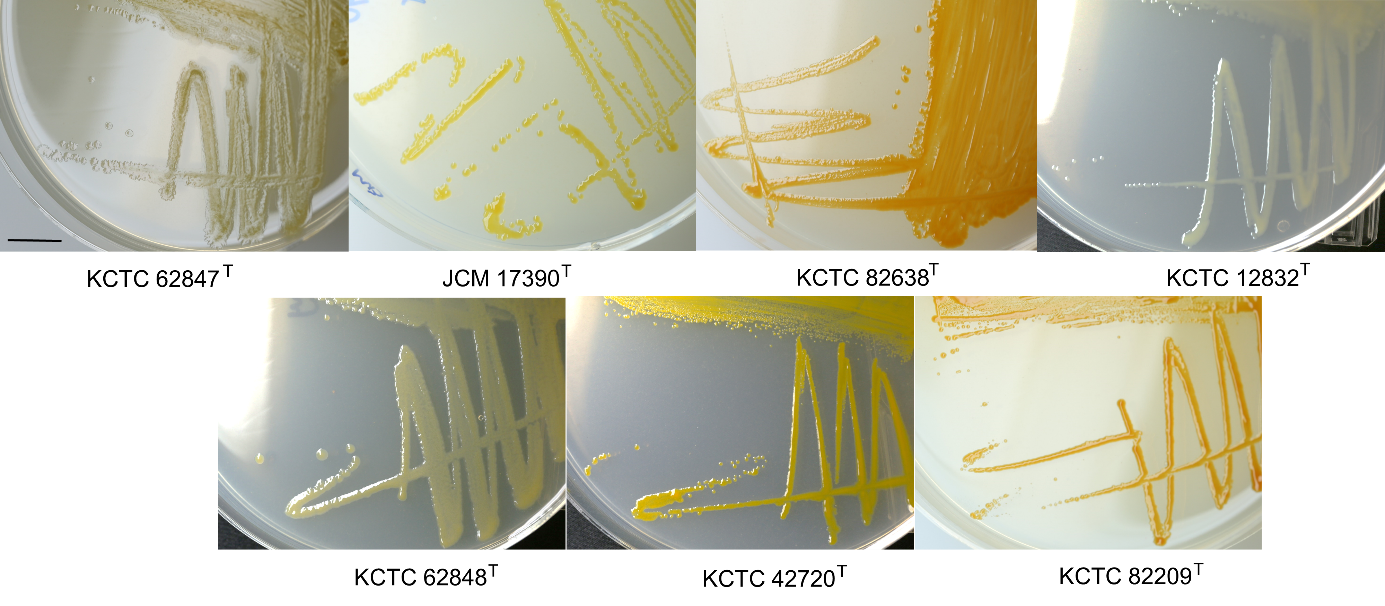


**Figure S1.** Colony morphology of existing members of genus *Fulvivirga.*

Taxa: *F. sediminis* 2943^T^ (KCTC 62847^T^); *F. imtechensis* JCM 17390^T^; *F. aurantia* KCTC 82638^T^; *F. kasyanovii* KCTC 12832^T^; *F. marina* 29W222^T^ (KCTC 62848^T^); *F. lutimaris* KCTC 42720^T^; *F. lutea* S481^T^ (KCTC 82209^T^). 4-day old plate. Scale bar: 1 cm.


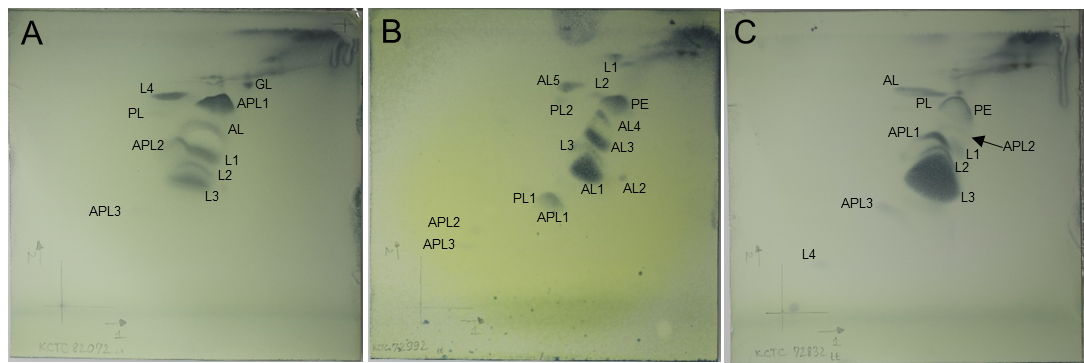


**Figure S2**. Polar lipid profiles of SS9-22^T^ (A), W9P-11^T^ (B), SW1-E11^T^ (C). AL: unidentified aminolipid, APL: unidentified aminophospholipid, PE: phosphatidylethanolamine, PL: unidentified phospholipid, GL: unidentified glycolipid, L: unidentified lipid.


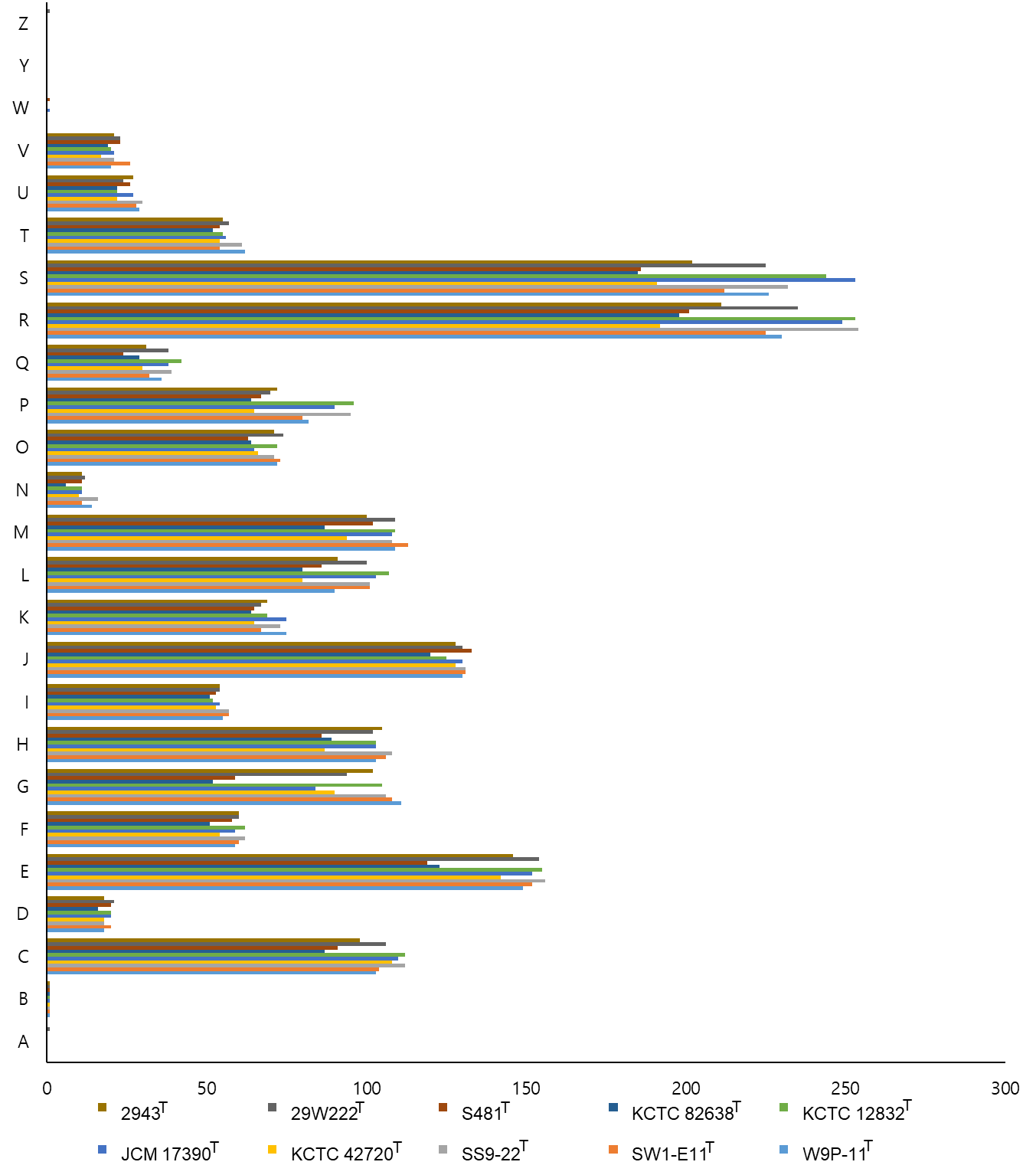


**Figure S3.** Comparison of clusters of orthologous groups (COGs) based on the COG database using RPS-BLAST (https://blast.ncbi.nlm.nih.gov/Blast.cgi) integrated in WebMGA (<https://github.com/weizhongli/webMGA>) of SS9-22^T^, W9P-11^T^, and SW1-E11^T^ with the existing species in the genus *Fulvivirga: F. sediminis* 2943^T^; *F. imtechensis* JCM 17390^T^; *F. aurantia* KCTC 82638^T^; *F. kasyanovii* KCTC 12832^T^; *F. marina* 29W222^T^; *F. lutimaris* KCTC 42720^T^; *F. lutea* S481^T^. A: RNA processing and modification; B: Chromatin structure and dynamics; C: Energy production and conversion; D: Cell cycle control, cell division, chromosome partitioning; E: Amino acid transport and metabolism; F: Nucleotide transport and metabolism; G: Carbohydrate transport and metabolism; H: Co-enzyme transport and metabolism; I: Lipid transport and metabolism; J: Translation, ribosomal structure and biogenesis; K: Transcription; L: Replication, recombination and repair; M: Cell wall/membrane/envelope biogenesis; N: Cell motility; O: Posttranslational modification, protein turnover, chaperones; P: Inorganic ion transport and metabolism; Q: Secondary metabolite biosynthesis, transport and catabolism; R: General function prediction only; S: Function unknown; T: Signal transduction mechanisms; U: Intracellular trafficking, secretion, and vesicular transport; V: Defense mechanisms; W: Extracellular structures; Y: Nuclear structure; Z: Cytoskeleton.
